# Harnessing desert resources: a comparative study of microgreens growth, nutrient dynamics, and performance in desert sand and rockwool

**DOI:** 10.3389/fpls.2025.1677009

**Published:** 2025-10-02

**Authors:** Drishya Nishanth, Chythra Somanathan Nair, Ramya Manoharan, Radhakrishnan Subramanian, Zienab F. R. Ahmed, Abdul Jaleel

**Affiliations:** ^1^ Department of Integrative Agriculture, College of Agriculture and Veterinary Medicine, U.A.E. University, Al Ain, United Arab Emirates; ^2^ ASPIRE Research Institute for Food Security in the Drylands (ARIFSID), United Arab Emirates University, Al Ain, United Arab Emirates

**Keywords:** microgreens, desert sand, nutritional profile, sustainability, food security

## Abstract

Food security in arid regions, such as the United Arab Emirates (UAE), is a significant challenge due to harsh environmental conditions such as low rainfall, high temperatures, and low-quality soil. These factors limit agricultural productivity and compromise stability. In such regions, where desert dunes are abundant, using sand as a plant growth medium presents unique opportunities to reduce reliance on expensive, synthesized substrates like rockwool, which have a larger carbon footprint. Using desert sand as an alternative to growing low-input, yet nutrient-rich crops, such as microgreens, offers a promising solution for sustainable food production in arid regions. This study aimed to assess the feasibility and effectiveness of using desert sand as a growth medium for cultivating six different types of microgreens in comparison to conventional rockwool medium (control), with a focus on their growth performance and nutritional quality. Six microgreens—Fenugreek (*Trigonella foenum-graecum* L.), Mustard (*Brassica juncea* L.), Ajwain (*Trachyspermum ammi* L.), Alfalfa (*Medicago sativa* L.), Arugula (*Eruca sativa* L.), and Thyme (*Thymus vulgaris* L.)—were grown in two different media: desert sand and rockwool (control). Growth parameters (shoot length, root development), yield (fresh and dry weight), and nutritional attributes (chlorophyll, carotenoids, total phenols, ascorbic acid, and antioxidant activity) were measured and compared across treatments. The results demonstrated that microgreens grown in desert sand outperformed those grown in rockwool, exhibiting longer shoot lengths, increased root development, and higher yields. Alfalfa and fenugreek recorded the highest fresh and dry weights. Additionally, the type of media had a significant impact on the phytochemical content. Microgreens grown in sand showed higher levels of total phenols, antioxidants, and ascorbic acid—particularly in ajwain and thyme—while chlorophyll and carotenoid content showed minor variations across both media. The superior performance of microgreens in desert sand can be attributed to the mineral content of sand, especially calcium, as confirmed by ICP-MS analysis. Desert sand emerges as a sustainable and cost-effective alternative to non-biodegradable substrates like rockwool, offering a viable solution for the cultivation of nutrient-rich microgreens in arid regions.

## 1 Introduction

Food insecurity, as defined by the Food and Agriculture Organization (FAO), is the lack of a reliable supply of nutritious and safe food essential for fostering development, supporting growth, and promoting a healthy and active lifestyle. In 2019, nearly one in ten people worldwide experienced severe food insecurity. Recent estimates indicate that over 8.9% of the global population suffers from hunger, marking an increase of more than 10 million people in the past year and nearly 60 million over the last five years (Bongaarts, 2021). This growing food insecurity is partly due to rapid industrialization and urbanization, which have led to increased greenhouse gas emissions, negatively impacting agricultural production and reducing the amount of arable land. To secure food supplies for the ever-growing global population, it is crucial to explore and develop alternative sustainable food production methods that are more efficient, productive, and environmentally friendly than traditional farming practices (Napoli et al., 2011; FAO, 2022). Urban farming, recognized as a highly efficient solution, involves a range of techniques such as vertical farming, greenhouses, aquaponics, hydroponics, and more (Benke and Tomkins, 2017). Data analytics and machine learning are employed to monitor, optimize, and potentially automate crop cultivation within controlled environments. Such indoor farming methods offer potential advantages in terms of convenience and environmental sustainability, particularly in arid regions where water availability is minimal. Despite the attention it receives, controlled environment agriculture (CEA) is currently limited in its applicability to certain crops. However, microgreen cultivation is emerging as a particularly notable aspect within this realm due to its positive impact on the environment and economic feasibility (Singh et al., 2024). Microgreens, characterized by their exceptionally high nutritional content, reportedly up to 40 times more than mature vegetables, are rapidly gaining popularity within controlled environments (Lone and Pandey, 2024). Microgreens, often referred to as ‘baby greens’, are young vegetable greens that have advanced beyond the sprout stage but have not yet developed their true leaves. These young, tender greens, derived from the seeds of various herbs, vegetables, and grains, consist of a central stem and include two sets of immature true leaves (Di.M.C. Bella et al., 2020; Ghoora et al., 2020). Depending on the species, microgreens typically reaches heights of 1 to 3 inches and are generally harvested between 7 to 21 days after sowing (Alhindaassi et al., 2025). Microgreens offer enhanced nutritional value, more intense flavor, and superior taste (Puccinelli et al., 2019). Studies show that microgreens may contain higher concentrations of phytochemicals, minerals, and vitamins than their fully mature counterparts (Yadav et al., 2019; Xiao et al., 2019).

Microgreens thrive in environments which maintain consistent temperature and humidity levels. A production area of 400 square meters (m²) can efficiently yield 90 kg of microgreens, corresponding to approximately 300 g/m^2^ yield per week (Lone and Pandey, 2024). Harvest time varies by species and growth conditions, with some microgreens ready in just 10–14 days. Temperature plays a significant role in the growth of microgreens, with optimal cultivation typically requiring temperatures below 20 °C. Research by Allred and Mattson (2018) indicated that increasing the production temperature from 14 °C to 22 °C leads to a linear reduction in yield by 35%–40%. Additionally, the choice of substrate for germination is critical in microgreens production. Substrates with a depth of 1.75–6 cm resulted in a higher fresh weight of harvested microgreens (Allred and Mattson, 2018). Various media types are commonly used for growing microgreens including sand, vermiculite composts, and composts made from peat, sand, coconut coir dust, sugarcane filter cake, and vermicompost (Anon, 2016; Xiao et al., 2019). Research by Muchjijab et al. (2015) demonstrated the effectiveness of these media for cultivating microgreens. However, conventional growth substrates like rockwool, perlite, vermiculite, and peat moss present challenges due to their high cost, non-biodegradability, and reliance on non-renewable resources. Rockwool demonstrates properties such as high porosity (92-97%) as peatmoss, and water retention characteristics resembling sand, making it suitable for the growth of microgreens (Acuna et al., 2004). Gruda (2019) emphasized the waste disposal problem associated with rockwool. Hashida et al. (2014) found that long-term rockwool use can significantly enhance nitrous oxide emissions, with environmental factors like water content strongly influencing these greenhouse gas emissions, raising further environmental concerns about rockwool’s sustainability in agricultural applications. Shifting toward sustainability is essential and hence desert sand may be a promising choice for the cultivation of microgreens.

In arid regions, desert sand, despite its low nutrient content, presents a potentially cost-effective and environmentally sustainable alternative medium for cultivating microgreens.This is largely because the inherent nutritional reserves within microgreen seeds often minimize or eliminate the need for additional fertilizers during production (Sangwan et al., 2022). Limited experimental evidenceexists to supportthe use of desert sand as a growth substrate for microgreens. The present study investigates the cultivation of six microgreen species—Fenugreek (*Trigonella foenum-graecum* L.), Mustard (*Brassica juncea* L.), Ajwain (*Trachyspermum ammi* L.), Alfalfa (*Medicago sativa* L.), Arugula (*Eruca sativa* L.), and Thyme (*Thymus vulgaris* L.)—using desert sand, to evaluate its feasibility. The primary objective was to compare growth parameters, yield, and nutritional quality of microgreens grown in desert sand with those cultivated in a conventional substrate such as rockwool.

## 2 Materials and methods

### 2.1 Collection of desert sand

Desert sand was collected from three different locations in Al Ain,located in the eastern region of the emirate of Abu Dhabi (24.2232° N, 55.7229° E), (the United Arab Emirates (UAE). The samples were transferred to plastics bags and filtered using a 200 mm mesh sieve. The physico-chemical properties such as bulk density, water holding capacity, pH, electrical conductivity (EC) and salinity of desert sand were measured according to Poudel et al. (2023). The nutrient and mineral content of sand was determined by the ICP-MS (Inductively Coupled Plasma-Mass Spectrometer) method as outlined by Suleman and Elagib (2015).

### 2.2 Seed material for microgreens production

Seeds of the six species: Fenugreek (*Trigonella foenum-graecum* L.), Mustard (*Brassica juncea*), Ajwain (*Trachyspermum ammi* L.), Alfalfa (*Medicago sativa* L.), Arugula (*Eruca sativa* L.), and Thyme (*Thymus vulgaris* L), were procuredfrom certified local dealers. Seeds procured had a germination rate of over 80% and were free from any seed treatment.

### 2.3 Microgreens cultivation and growing conditions

**Figure 1:**
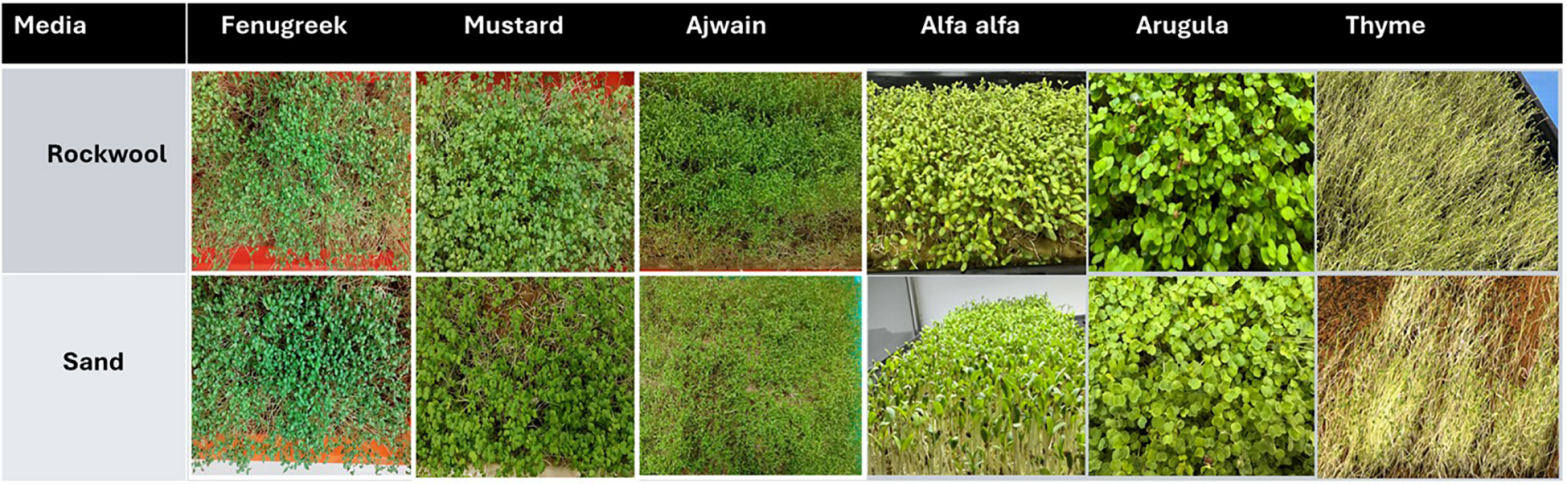
Visual comparison of microgreens cultivated in rockwool and desert sand media.

Microgreen production was done indoors and a temperature of 22–28 °C and a relative humidity ranging between 60-70% was maintained. The plants received indirect sunlight as they were placed near a south-facing window. South-facing windows typically receive higher light exposure compared to other orientations, but the intensity can vary depending on factors such as time of day, season, and any shading from nearby structures. Based on typical indoor conditions, the light intensity at the window surface may range from 200 to 600 μmolm^-^²s^-^¹ during daylight hours, which is sufficient for microgreen growth. According to literature, the most frequently used photosynthetic photon flux density (PPFD) of light intensities ranges from 100 to 300 µmol·m^−2^·s^−1^ for the growth of microgreens (Gao et al., 2021). A uniform seed rate of 30 g per tray was used for all species. Seeds were soaked in distilled water overnight andsown in seedling trays of cell size 50 cm x 25 cm x 5 cm. The substrate for each microgreen consisted of rockwool (control) and desert sand (treatment) withthree replicates per treatment. Agrowth medium layer of approximately 5 cm thickness was usedfor each crop species (Sangwan et al., 2022). The soaked seeds were evenly spread on the growth media on trays. The trays were then covered with a lid until germination occurred.A bottom watering method was used to water the seedlings. Water sprays were also given twice a week. Each microgreen species was harvested separately between the 3^rd^ and 21^st^ days after sowing (DAS) based on their two true leaf stage (**Figure 1**). No additional nutrients were added. Harvesting themicrogreens was done by trimming the stems at about 1.0 cm above the substrateusing sanitized scissors.

### 2.4 Growth parameters

Data on growth parameters such as root length, shoot length, yield, fresh and dry weight, and moisture content were collected on the day of harvesting for each treatment and replicate, random sampling was done to collect a sample size of 20 plants from the middle of the seedling trays using a spatula. The seedlings were carefully placed on tissue paper, and the loosely attached media was removed from the roots. The roots were then thoroughly washed with deionized water with a minimum root loss of < 3%. After blotting to remove excess water, root and shoot lengths were measured, and fresh weight was recorded. Dry weight was determined after oven-drying the samples at 65 °C until constant weight. Moisture content (%) was calculated as:


Moisture content (%)=(Fresh weight–Dry weight/Fresh weight)×100

### 2.5 Estimation of total chlorophyll content and carotenoid

A direct spectroscopic method was employed for determining the total chlorophyll content (TCC) in the microgreen plant samples. About1g of each microgreen sample was added to 10 mL of 80% (v/v) aqueous acetone solution and then centrifuged at 5000 rpm for 5 minutes. The resulting supernatant was diluted to a final volume of 25 mL with 80% acetone. Absorbance was read at 646, 663, and 710 nm using a UV-Visible spectrophotometer, with 80% acetone serving as the blank. TCC was then calculated using the following equation (Xiao et al., 2014):

$\text{TCC }\left(\text{mg}/\text{g FW}\right)=\left[\left({\text{A}}_{646\;nm}–{\text{A}}_{710}nm\right)\times 0.01732+\left({\text{A}}_{663\;nm}–{\text{A}}_{710}nm\right)\times 0.00718\right]\times DF/FW$ where;

- A_646_,A_663_, A_710_ = absorbance values at 646, 663, and 710 nm, respectively
- DF = dilution factor
- FW = fresh weight of the sample (g)

Carotenoid content was estimated using the formula and expressed in milligrams per gram fresh weight at absorbances 480, 663 and 663 nm (Kirk and Allen, 1965). $\text{Carotenoids }\left(\text{mg}/\text{g FW}\right)={\text{A}}_{480\;nm}+\left(0.114\times {\text{A}}_{663\;nm}–0.638\times {\text{A}}_{645\;nm}\right)/\text{FW}$ where;

- A_480 nm_,A_645 nm_,A_663 nm_ = absorbance values at respective wavelengths
- FW = fresh weight of the sample (g)

### 2.6 Phenolic content and antioxidant activity

#### 2.6.1 Preparation of methanolic extract

To prepare the methanolic extract, 0.2 g of fresh microgreens were weighed and homogenized in 20 mL of 80% methanol for 30 seconds. From this mixture, 1.0 mL of the aliquot was taken, combined with 0.4 mL of hexane to remove chlorophyll, and homogenized for another 30 seconds. The sample was then centrifuged at 5000 rpm for 10 minutes at 25 °C. The supernatant was discarded, and the extract was washed twice with hexane and dried. Finally, the dry weight of the extract was dissolved in 1 mL of 80% methanol (Lester et al., 2013).

#### 2.6.2 Total phenolic content

Microgreen samples were analysed for total phenolic content using chlorophyll-free methanolic extracts, following the Folin–Ciocalteu (FC) method as described by Niroula et al. (2019). About 0.5 mL of the sample extract was mixed with 2.0 mL of 10% F-C reagent and left at room temperature for approximately 10 minutes. Following this, 2.0 mL of sodium carbonate (7.5%, w/v) reagent was added and thoroughly mixed. Subsequently, 2.0 mL of distilled water was added, and the mixture was incubated at 40 °C for 45 minutes. After incubation, the mixture was returned to room temperature and absorbance was measured at 765 nm using a spectrophotometer. Gallic acid was used as the standard a calibration curve was drawn using concentrations between 1 mg/mL and 10 mg/mL. The results were expressed as gallic acid equivalents (GAE).

#### 2.6.3 DPPH radical scavenging activity

Radical scavenging activity was performed using 2,2-diphenyl-1-picrylhydrazyl (DPPH) and the methanolic sample extract following the modified method of Yu et al. (2002). About 300 µL of de-chlorophylated plant extract was added to 500 µL of 0.15 mM DPPH in methanol solution. The samples were incubated in the dark for 30 minutes, and the absorbance was measured at 517 nm against 80% (v/v) methanol as the blank, with DPPH in methanol used as the positive control. Radical scavenging activity (inhibition%) was calculated using the standard formula.

### 2.7 .Total protein

The total protein content was measured using the Bradford assay, following the modified method of Hammond and Kruger (1988) usings odium phosphate. About0.2 g of the microgreen plant tissue samples were ground with 5 mL of ice-cold extraction buffer containing 50 mM of sodium phosphate buffer at pH 7.0 and 0.1mN EDTA. The sample mixtures were vortexed and centrifuged at 15,000 rpm for 20 minutes. The pellets were discarded while the supernatant was collected and stored in ice. The supernatant was mixed with 100 µL of enzyme extract and 1 mL of Bradford reagent and incubated for 5 minutes. Absorbance was read at 595 nm against Bradford reagent as the blank. The protein concentration was determined from the standard curve using BSA (bovine serum albumin) as a standard at different concentrations.

### 2.8 Ascorbic acid

Ascorbic acid content was evaluated using the method as described by Xiao et al., 2014. Briefly, 1.0 g of fresh microgreen samples was macerated in 10 mL of 5% cooled metaphosphoric acid (w/v) solution, prepared in 10% acetic acid (v/v). The mixture was vortexed at room temperature for 5 minutes and then centrifuged at 8000 rpm for 10 5minutes at 4 °C. The supernatant was collected and filtered through Whatman No.1 filter paper. For the ascorbic acid assay, a reaction mixture was prepared by combining 2.0 mL of the sample extract with 1.0 mL of 2,6-dichlorophenol indophenols (0.02% w/v), 2.0 mL of thiourea (2% w/v), and 1.0 mL of 2,4-dinitrophenylhydrazine (2% in 5M H_2_SO_4_). The mixture was thoroughly mixed and incubated at 50 °C for 60 minutes with occasional stirring. The reaction mixture was then placed on ice to maintain a temperature of 4 °C. Following this, 4.0 mL of 85% H_2_SO_4_ was slowly added with gentle shaking, and the mixture was incubated for an additional 30 minutes. The absorbance of the final mixture was measured at 520 nm. Standard curves were prepared using various concentrations of ascorbic acid.

### 2.9 Elemental analysis

The simultaneous analysis of major minerals and trace elements in plant samples was conducted using Inductively Coupled Plasma Optical Emission Spectroscopy (ICP-OES). Element extraction from the plant samples was achieved using the CEM Mars 5 microwave digestion system, in accordance with USEPA method 3015A (Nishanth et al.,2024). This method mimics traditional extraction with nitric acid (HNO_3_) and hydrochloric acid (HCl) through heating. For sample preparation, 0.5 gr of each plant sample was accurately weighed and placed into microwave digestion vessels, to which 10 mL of concentrated nitric acid and 2 mL of hydrochloric acid were added. The vessels were then sealed and placed in the microwave digestion system. ICP Expert software was employed to set up an appropriate program, selecting the analyte elements along with their wavelengths, sensitivities, interferences, and linear regression equations. Mixed calibration standard solutions (Merck) were used to create calibration curves for each element, and calibration check standard solutions (Romil) were also aspirated and their values recorded. Finally, the prepared sample solutions were aspirated, and element concentrations (P, K, Ca, Mg, S, Na, Fe, Mn and Zn) were determined using the calibration curves.

### 2.10 Statistical analysis

Analysis of all samples was conducted in triplicate and expressed as mean ± standard deviation. A comparison was made between the two substrates for each microgreen species. Statistical analysis was performed using Minitab software. A two-way ANOVA, followed by Tukey’s HSD test, was employed to compare the means. Statistical significance was determined at a p-value of ≤ 0.05.

## 3 Results

### 3.1 Physico-chemcial properties of desert sand

**Table 1** summarizes the physiochemical properties of desert sand along with a comparison with rockwool. Desert sand exhibited a bulk density of 1.28 ± 0.07 g/cm³, which was considerably higher than that of rockwool (0.067 ± 0.01 g/cm³), reflecting its denser structure. The water holding capacity of sand was low (6.44 ± 1.14%) compared to rockwool (83.41 ± 2.41%), indicating limited water retention. The pH of sand was slightly alkaline (7.21 ± 1.18), while rockwool was slightly acidic (6.83 ± 0.29). Electrical conductivity (EC) and salinity were higher in sand (1.93 ± 0.33 mS/cm and 1228.33 ± 258.67 mg/L, respectively) than in rockwool (0.53 ± 0.21 mS/cm and 101.67 ± 38.80 mg/L), suggesting greater ionic content in the natural substrate.

**Table 1:** Physico-chemical properties of collected sand samples and rockwool.

Physicochemical properties	Desert sand	Rockwool
Bulk density	1.28± 0.07	0.067 ± 0.01
Water Holding Capacity (%)	6.44 ± 1.14	83.41 ± 2.41
pH	7.21 ± 1.18	6.83 ± 0.29
EC (mS/cm)	1.93 ± 0.33	0.53 ± 0.21
Salinity (mg/L)	1228.33 ± 258.67	101.67 ± 38.80

Analysis of all samples was conducted in triplicates and expressed as mean ± standard deviation.

### 3.2 Elemental analysis of desert sand

The elemental analysis of desert sand was done using ICP-MS and the results are summarized in **Table 2**. Among macronutrients, calcium was most abundant (5801.07 ± 100.1 mg/kg), followed by magnesium (874.47 ± 5.1 mg/kg), potassium (148.58 ± 0.8 mg/kg), phosphorus (115.52 ± 1.94 mg/kg), sulfur (281.95 ± 6.55 mg/kg), and sodium (289.31 ± 3.07 mg/kg). Micronutrients were present in lower concentrations, including iron (726.49 ± 7.55 mg/kg), zinc (24.34 ± 0.25 mg/kg), manganese (15.11 ± 0.5 mg/kg), and copper (9.15 ± 2.11 mg/kg). Trace elements such as cobalt, nickel, chromium, molybdenum, lead, arsenic, and vanadium were detected at very low concentrations, while cadmium and arsenic were below detectable limits (<0.001 mg/kg and<0.009 mg/kg, respectively).

**Table 2:** Elemental composition of desert sand.

Element	Value (mg/kg (ppm))
P	115.52 ± 1.94
K	148.58 ± 0.8
Ca	5801.07 ± 100.1
Mg	874.47 ± 5.1
S	281.95 ± 6.55
Na	289.31 ± 3.07
Fe	726.49 ± 7.55
Mn	15.11 ± 0.5
Zn	24.34 ± 0.25
Cu	9.15 ± 2.11
Co	5.36 ± 1.23
Ni	142.70 ± 0.3
Cr	64.12 ± 2.02
Cd	<0.001
Al	147.79 ± 10.5
Mo	<0.018
Pb	1.93 ± 0.09
As	<0.009
Sr	238.12 ± 0.82
V	14.24 ± 3.5

Analysis of all samples was conducted in triplicates and expressed as mean ± standard deviation.

### 3.3 Growth parameters

**Figure 2:**
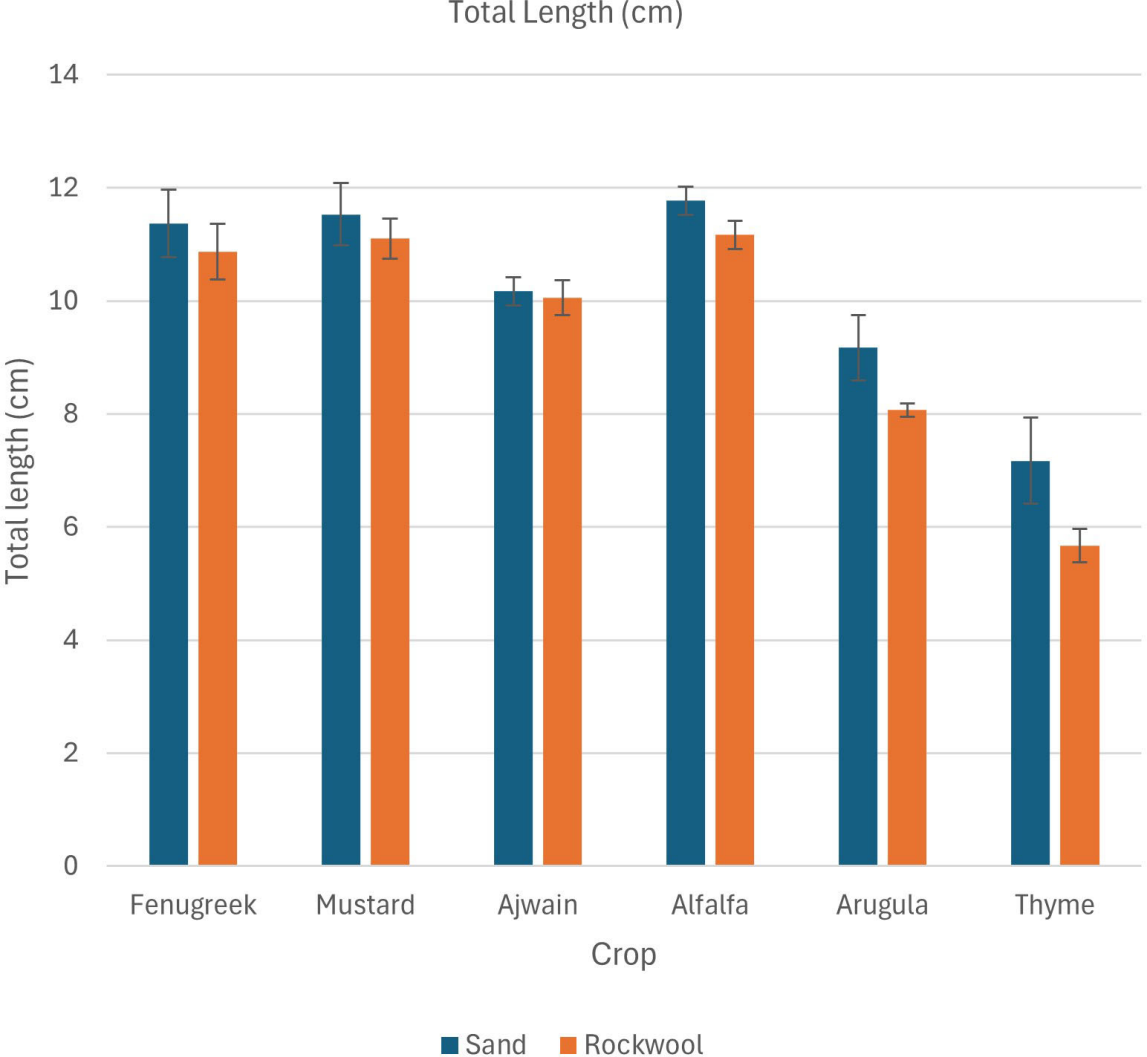
Total length (cm) of different microgreen species in sand and rockwool media. ANOVA with Tukey’s post hoc test was performed (p < 0.05). Different superscripts indicate significant differences; absence of superscripts indicates no significant differences.

The findings revealed that both crop species and media type influenced the total length of microgreens. Microgreens grown in desert sand generally showed greater total length compared to those grown in rockwool. However, the effect of the growth medium on total plant length was consistent across all crop species, with no significant variation in its impact among the different species. Alfalfa exhibited the longest total length (**Table 3**; **Figure 2**) in both desert sand (11.77 cm) and rockwool (11.17 cm) followed by mustard and fenugreek.

**Table 3:** Growth parameters of the different microgreen species when grown in sand and rockwool media.

Parameters	Fenugreek		Mustard		Ajwain		Alfalfa		Arugula		Thyme	
	Sand	Rockwool	Sand	Rockwool	Sand	Rockwool	Sand	Rockwool	Sand	Rockwool	Sand	Rockwool
Total Length (cm)	11.37 ± 0.60	10.87 ± 0.49	11.53 ± 0.55	11.10 ± 0.36	10.17 ± 0.25	10.06 ± 0.31	11.77 ± 0.25	11.17 ± 0.25	9.17 ± 0.58	8.07 ± 0.12	7.17 ± 0.76	5.67 ± 0.29
Shoot Length (cm)	7.83 ± 0.55	7.66 ± 0.09	7.67 ± 0.33	7.60 ± 0.12	7.79 ± 0.17	7.67 ± 0.18	6.83 ± 0.58	6.29 ± 0.29	5.17 ± 0.76	4.83 ± 0.35	4.60 ± 0.85	3.50 ± 0.50
Root Length (cm)	3.53 ± 0.74	3.20 ± 0.41	3.86 ± 0.87	3.50 ± 0.47	2.38 ± 0.24	2.39 ± 0.19	4.93 ± 0.60	4.87 ± 0.65	4.00 ± 0.50	3.23 ± 0.45	2.57 ± 1.01	2.17 ± 0.58
Yield/Tray Fresh Weight (g)	232.33 ± 7.51^a^	163.67 ± 11.93^d^	197.67 ± 9.50^bc^	120.33 ± 3.51^e^	193.33 ± 6.66^bc^	174 ± 5.57^cd^	218.33 ± 7.64^ab^	191.33 ± 10.02^c^	116.67 ± 7.64^e^	102.33 ± 6.43^ef^	91.00 ± 11.53^fg^	68.67 ± 11.02^g^
Dry Weight (g)	21.2 ± 0.40^b^	10.54 ± 0.21^d^	16.25 ± 0.97^c^	9.52 ± 0.49^de^	10.38 ± 0.13d	7.97 ± 0.17^e^	25.60 ± 1.22^a^	15.10 ± 0.36^c^	14.80 ± 0.92^c^	9.90 ± 0.82^d^	10.99 ± 0.32^d^	7.96 ± 0.56^e^
Moisture Content (%)	90.84 ± 0.20	93.54 ± 0.46	91.75 ± 0.48	92.08 ± 0.63	92.74 ± 0.49	93.53 ± 0.60	88.27 ± 0.40	92.10 ± 0.34	87.26 ± 1.43	90.31 ± 0.63	87.81 ± 1.36	88.23 ± 1.75
Harvest time (days)	13.67 ± 0.58^ef^	11.68 ± 0.58^f^	17.68 ± 0.58^bc^	14.68 ± 0.58^de^	20.68 ± 0.58^a^	16.3 ± 0.58^cd^	14.33 ± 0.58^de^	13.67 ± 0.58^ef^	15.00 ± 1.00^de^	15.33 ± 0.58^de^	21.33 ± 0.58^a^	19.67 ± 0.58^ab^

Values are given as mean ± SD of 20 samples replicated thrice in each group. Values with no superscripts showed no interaction between the factors during the 2-way ANOVA analysis. Values that do not share a common superscript (a,b,c,d,e,f,g) differ significantly at P ≤ 0.05 (Tukey’s HSD test).

The findings indicated that both media type and crop species significantly influenced shoot length. Microgreens grown in sand generally exhibited longer shoots compared to those grown in rockwool, and significant differences were observed among the different crop species. The interaction between media and crop species was not significant, suggesting that the effect of media on shoot length was consistent across all crops studied. Fenugreek recorded the highest shoot length under both sand (7.83 cm) and rockwool (7.66 cm) media. Mustard and ajwain exhibited comparable shoot lengths across both growth media. The lowest value for shoot length was recorded in thyme grown under both sand (4.60 cm) and rockwool (3.50 cm).

The findings showed that root length varied significantly among different crop species, while the type of media (desert sand *vs*. rockwool) had no noticeable impact. Furthermore, there was no interaction between crop species and media, meaning the media’s influence on root length was consistent across all species. The average root length varied across microgreens and substrates, with higher root length observed in sand compared to rockwool (**Table 3**). Among the microgreens, alfalfa recorded the highest value (4.93 cm in sand, 4.87 cm in rockwool), followed by mustard (3.86 cm in sand, 3.50 cm in rockwool) and arugula (4.00 cm in sand, 3.23 cm in rockwool). Thyme exhibited the shortest root length in both growth media (2.57 cm in sand and 2.17 cm in rockwool).

**Figure 3:**
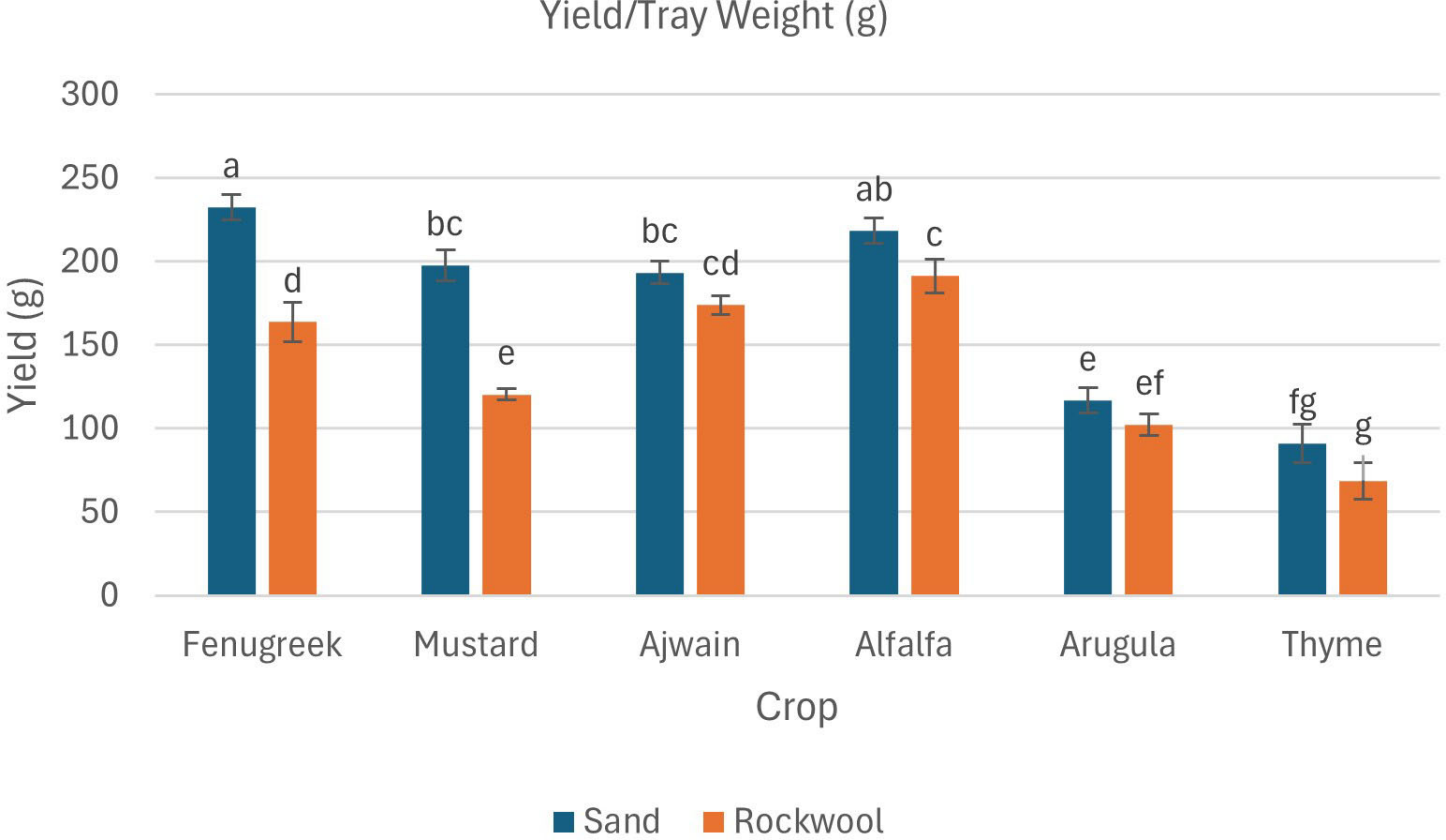
Yield/tray fresh weight (g) of different microgreen species under sand and rockwool media. ANOVA with Tukey’s post hoc test was performed (p < 0.05). Different superscripts indicate significant differences; absence of superscripts indicates no significant differences.

**Figure 4:**
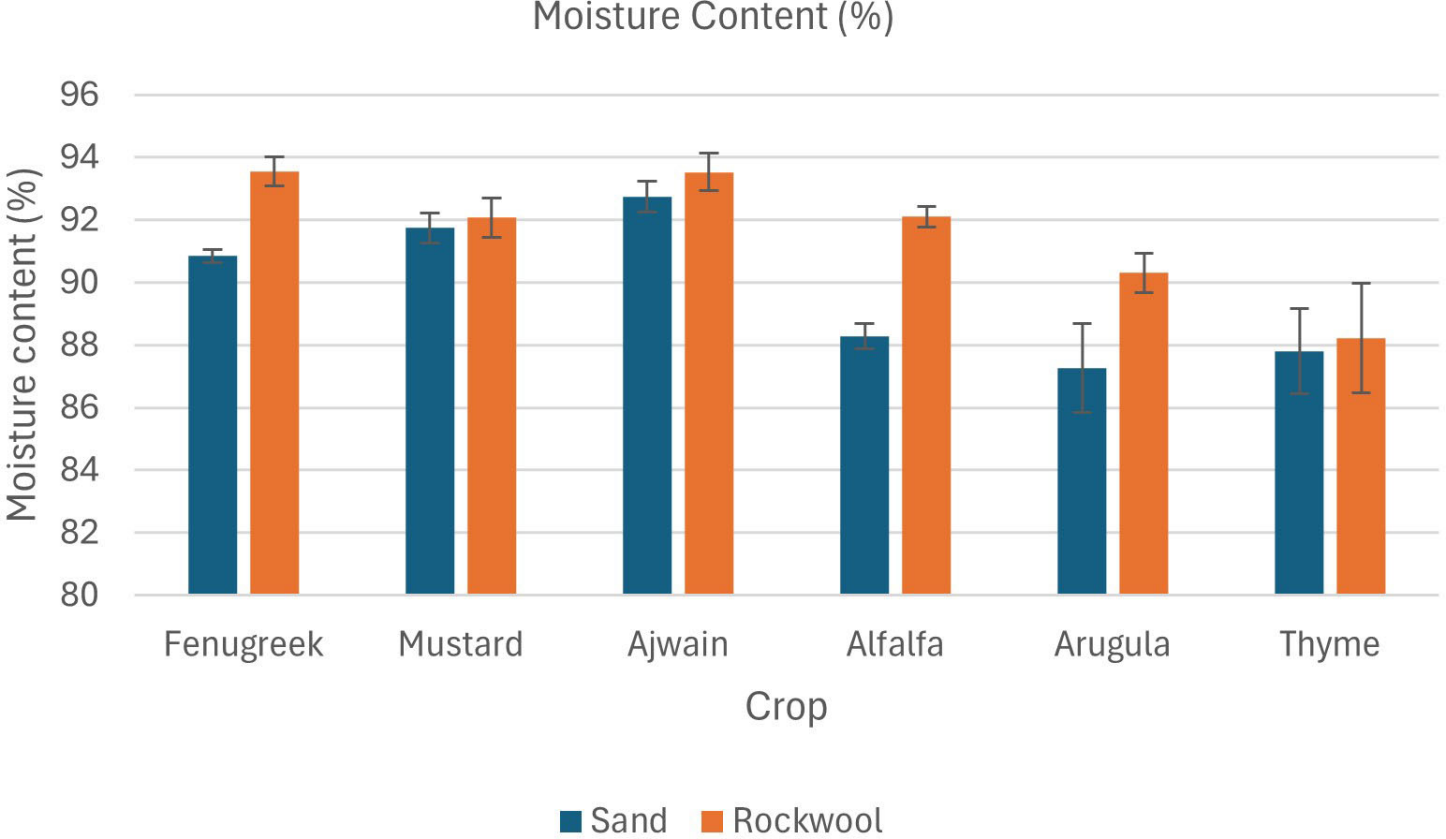
Moisture content (%) of microgreens as influenced by sand and rockwool growth media. ANOVA with Tukey’s post hoc test was performed (p < 0.05). Different superscripts indicate significant differences; absence of superscripts indicates no significant differences.

The data also revealed that both the crop species and the growth medium significantly affected fresh weight (**Table 3**; **Figure 3**). Additionally, there was a notable interaction between crop species and media, indicating that the effect of media on fresh weight varied depending on the species being grown. Fenugreek recorded the highest fresh weight (232.33 g) in sand, whereas alfalfa reached its maximum yield in rockwool (191.33 g). Thyme recorded the lowest fresh weight, with slightly better performance in sand (91 g) than in rockwool (68.67 g). Overall, sand consistently produced higher yields, with alfalfa, mustard, and fenugreek showing significantly greater fresh weight in this substrate, highlighting its potential as a superior growth medium. Substrate-related differences in moisture content for each crop are presented in **Figure 4**.

**Figure 5:**
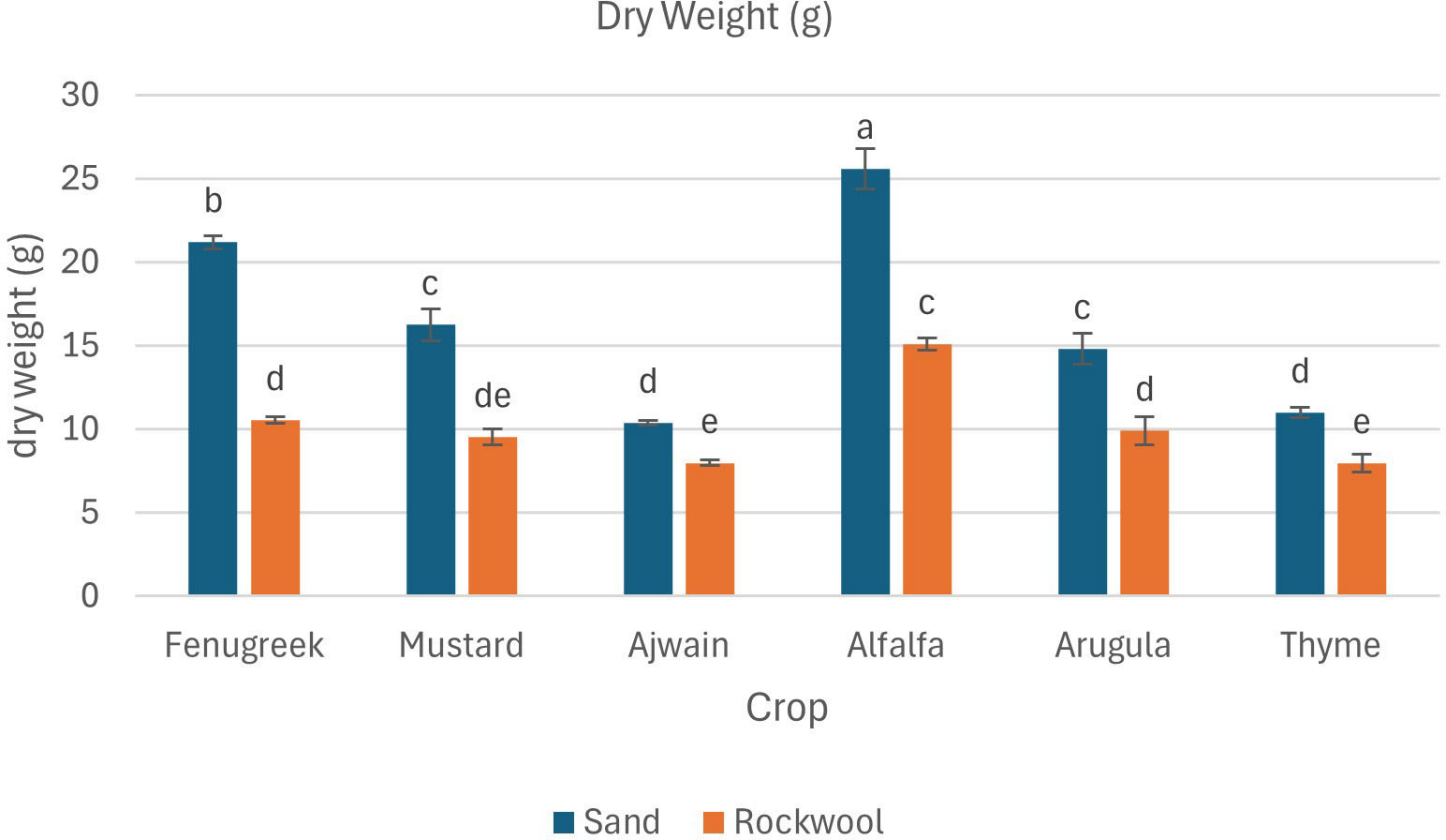
Dry weight (g) of different microgreen species under sand and rockwool media. ANOVA with Tukey’s post hoc test was performed (p < 0.05). Different superscripts indicate significant differences; absence of superscripts indicates no significant differences.

The results indicate that all crops exhibited significantly higher dry weight when grown in sand compared to rockwool. The highest dry weight was recorded for the alfalfa microgreens grown in sand (25.60 g) (**Figure 5**). The dry weight recorded was higher for all the six microgreen species grown in sand medium. The lowest dry weight was recorded for the thyme plants grown in rockwool medium (7.96 g). Microgreens cultivated in rockwool retained more moisture compared to those grown in sand. This was particularly notable in the case of fenugreek (93.54%) and ajwain (93.53%). The moisture content (%) for mustard plants remained consistent across both substrates which was similar with the thyme plants. The results indicate that both the media and crop species significantly influenced the moisture content of the microgreens. However, there was no significant interaction between media and crop species, suggesting that the impact of media on moisture content was consistent across all the crops studied.

Harvest time varied notably among the microgreens grown in sand and rockwool medium. Microgreens cultivated in desert sand exhibited prolonged maturation periods compared to those grown in rockwool (**Table 3**). Conversely, microgreens achieved maturity more rapidly when cultivated in rockwool. The results indicate that ajwain and mustard grown in rockwool had significantly shorter harvesting times compared to those grown in sand. Among the crops, fenugreek recorded the shortest harvest period of 12 days followed by alfalfa and arugula.

Overall, the yield and growth parameters of six microgreen species showed that desert sand can be used as a suitable growth medium for microgreen production.

### 3.4 Phytochemical analysis

The result of phytochemical analysis of different microgreens grown in sand and rockwool media are given in **Table 4**. Phytochemical analysis revealed varied responses of microgreens to different growth media. Across various parameters, sand media consistently demonstrated comparable or slightly higher phytochemical contents compared to rockwool media for all six microgreens: fenugreek, mustard, ajwain, alfalfa, arugula, and thyme.

**Table 4:** Phytochemical analysis of the different microgreen species in sand and rockwool media.

Parameters	Fenugreek		Mustard		Ajwain		Alfalfa		Arugula		Thyme	
	Sand	Rockwool	Sand	Rockwool	Sand	Rockwool	Sand	Rockwool	Sand	Rockwool	Sand	Rockwool
Total Chlorophyll (mg/g FW)	13.37 ± 0.93	13.60 ± 0.95	28.63 ± 1.58	29.57 ± 1.69	12.37 ± 4.91	10.70 ± 3.39	22.65 ± 1.33	22.40 ± 2.35	11.63 ± 3.19	11.27 ± 3.29	16.67 ± 2.52	15.67 ± 3.06
Carotenoid Content (mg/g FW)	0.93 ± 0.32^ab^	2.27 ± 1.02^ab^	2.57 ± 0.65^ab^	2.73 ± 0.40^a^	2.60 ± 0.79^a^	0.73 ± 0.45^b^	0.91 ± 0.28^ab^	0.96 ± 0.33^ab^	1.85 ± 0.74^ab^	1.68 ± 0.67^ab^	1.58 ± 0.29^ab^	1.93 ± 0.94^ab^
Total Phenol content (mg GAE/100g)	4.40 ± 0.60	1.10 ± 0.44	15.33 ± 4.15	12.30 ± 1.35	19.07 ± 0.67	16.27 ± 0.21	26.67 ± 2.52	23.17 ± 2.47	32.60 ± 1.51	27.57 ± 1.56	25.00 ± 2.65	21.63 ± 1.65
Total Antioxidant Activity (%)	13.20 ± 0.31	10.33 ± 0.49	22.70 ± 2.72	18.01 ± 0.78	28.05 ± 0.15	24.23 ± 0.42	58.04 ± 2.72	57.27 ± 2.61	31.07 ± 2.16	29.30 ± 2.52	27.45 ± 1.27	25.27 ± 0.64
Total Protein Content (mg/100g)	11.35 ± 0.64	11.10 ± 0.28	7.05 ± 0.10	7.43 ± 0.65	1.16 ± 0.10	1.24 ± 0.21	12.10 ± 0.36	11.79 ± 0.79	6.49 ± 0.62	6.33 ± 0.58	2.00 ± 0.85	2.10 ± 0.68
Ascorbic Acid Content (mg AA/100g of FW)	64.77 ± 1.23^c^	48.23 ± 1.17^ef^	51.80 ± 1.59^e^	43.17 ± 2.02^g^	72.73 ± 1.55^a^	68.37 ± 1.19^bc^	56.57 ± 1.16^d^	46.27 ± 1.42^fg^	37.40 ± 1.44^h^	34.63 ± 0.64^h^	70.12 ± 1.70^ab^	66.53 ± 1.79^bc^

Values are given as mean ± SD of 20 samples replicated thrice in each group. Values without superscripts indicate no significant interaction between the factors in the two-way ANOVA analysis. Values that do not share a common superscript (a, b, c, d, e, f) differ significantly (P ≤ 0.05, Tukey’s HSD test). Differences may be observed between species, substrates, or their interaction as assessed by two-way ANOVA (P ≤ 0.05, Tukey’s HSD test).

#### 3.4.1 Chlorophyll and carotenoid content

**Figure 6:**
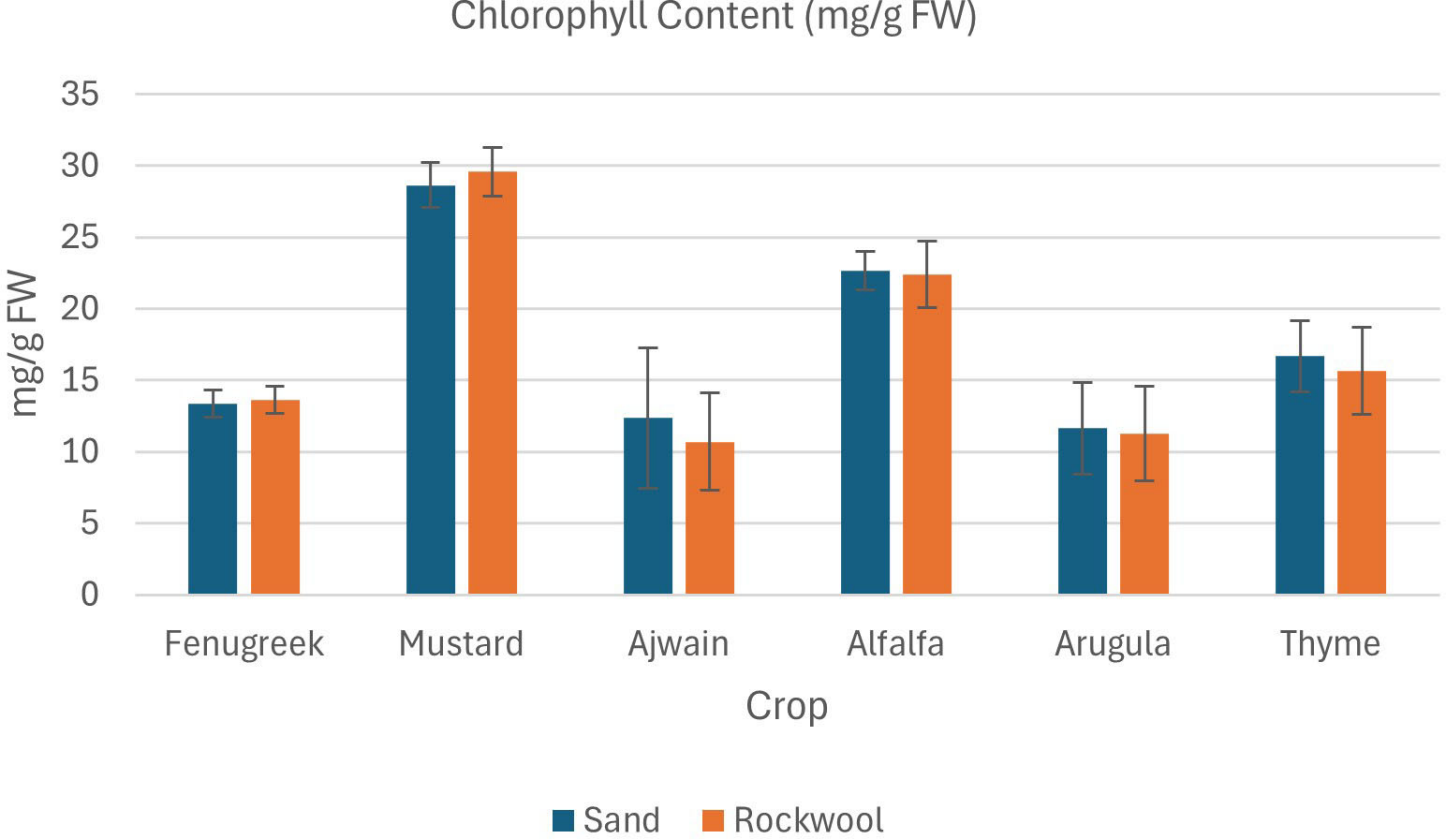
Chlorophyll content (mg/g FW) in different microgreen species grown under sand and rockwool media. ANOVA with Tukey’s post hoc test was performed (p < 0.05). Different superscripts indicate significant differences; absence of superscripts indicates no significant differences.

Microgreens in sand and rockwool medium showed similar trends in chlorophyll content, with minor variations observed in fenugreek. Mustard microgreens exhibited consistent chlorophyll levels across both growth media, while ajwain microgreens displayed higher differences in chlorophyll content between sand and rockwool (**Figure 6**). Mustard microgreens had the highest chlorophyll content across both substrates, followed by alfalfa. Ajwain plants recorded the lowest chlorophyll content across both substrates. The results indicate that crop species had a significant effect on chlorophyll content, with variations observed among the different crops. However, the type of media (sand *vs*. rockwool) did not have a significant impact on chlorophyll content, and there was no significant interaction between crop species and media, suggesting that the effect of media on chlorophyll content was consistent across all crops.

For carotenoids, most microgreens showed relatively consistent levels between sand and rockwool, indicating that the medium had minimal effect. Exceptions were fenugreek and ajwain. Fenugreek carotenoid content was higher in rockwool (2.27 mg/g FW) than in sand (0.93 mg/g FW), whereas ajwain exhibited higher carotenoids in sand (2.60 mg/g FW) compared to rockwool (0.73 mg/g FW). Other crops, including mustard, alfalfa, arugula, and thyme, displayed only minor differences between the two media. These results highlight that while sand generally favored microgreen growth and quality, certain species-specific traits, such as carotenoid accumulation in fenugreek and ajwain, deviated from this trend.

#### 3.4.2 Total phenol content

**Figure 7:**
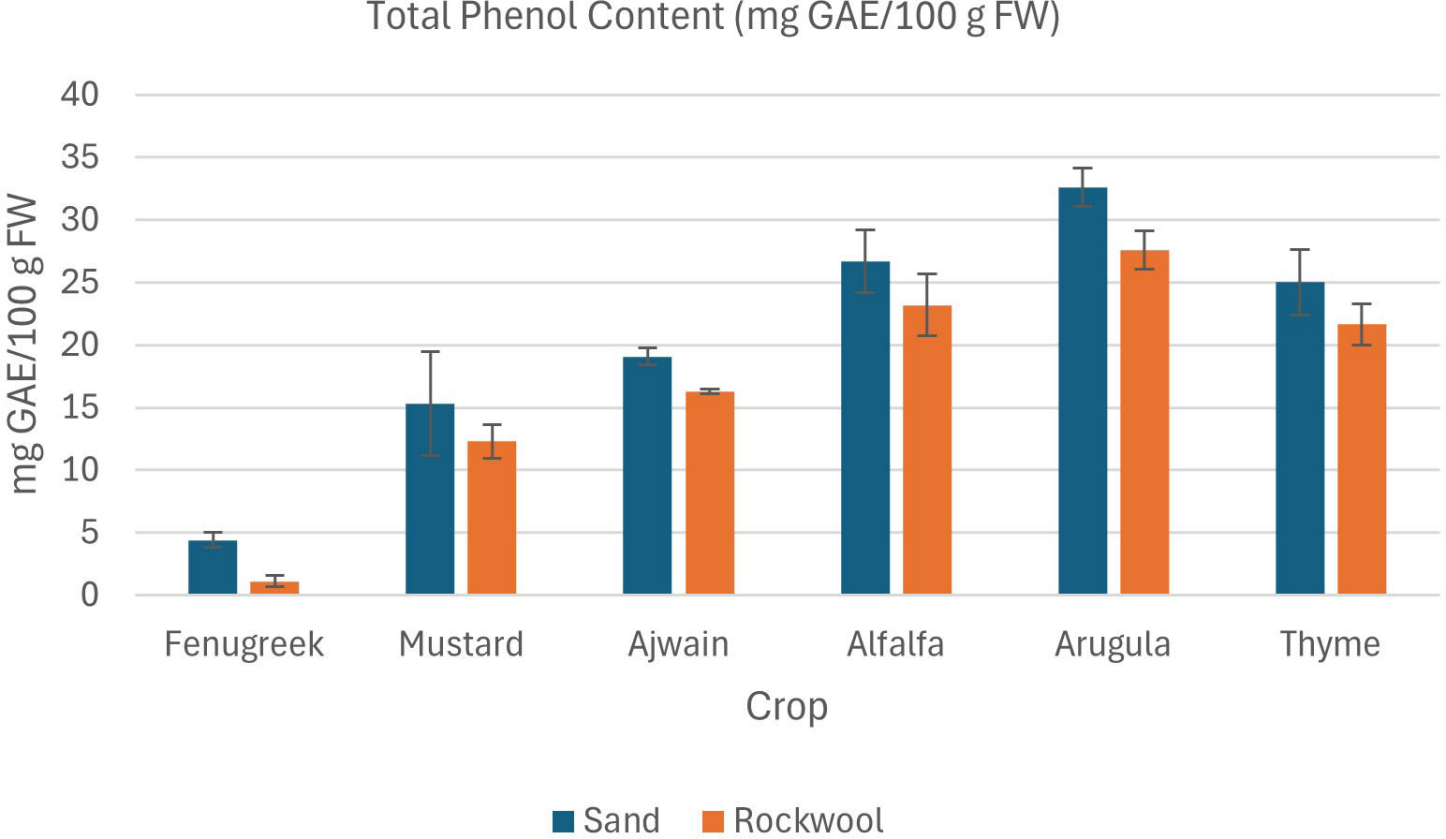
Total phenol content (mg GAE/100 g FW) in different microgreen species grown under sand and rockwool media. ANOVA with Tukey’s post hoc test was performed (p < 0.05). Different superscripts indicate significant differences; absence of superscripts indicates no significant differences.

Microgreens cultivated in sand demonstrated significantly higher total phenol content in comparison to those cultivated in rockwool (**Figure 7**). Among the crops, arugula consistently exhibited the highest phenol content (32.60 mg GAE/100 g) across both media types, while fenugreek displayed the lowest values rockwool (1.10 mg GAE/100 g), particularly in rockwool. Other crops, such as mustard and thyme, showed relatively consistent phenol levels, but still exhibited higher values in sand compared to rockwool. The findings show that crop species and the type of media (sand *vs*. rockwool) both had a notable impact on phenol content. The consistently elevated phenol content in sand-grown microgreens shows the potential of desert sand as a promising substrate for enhancing the nutraceutical quality of microgreens.

#### 3.4.3 Antioxidant content

**Figure 8:**
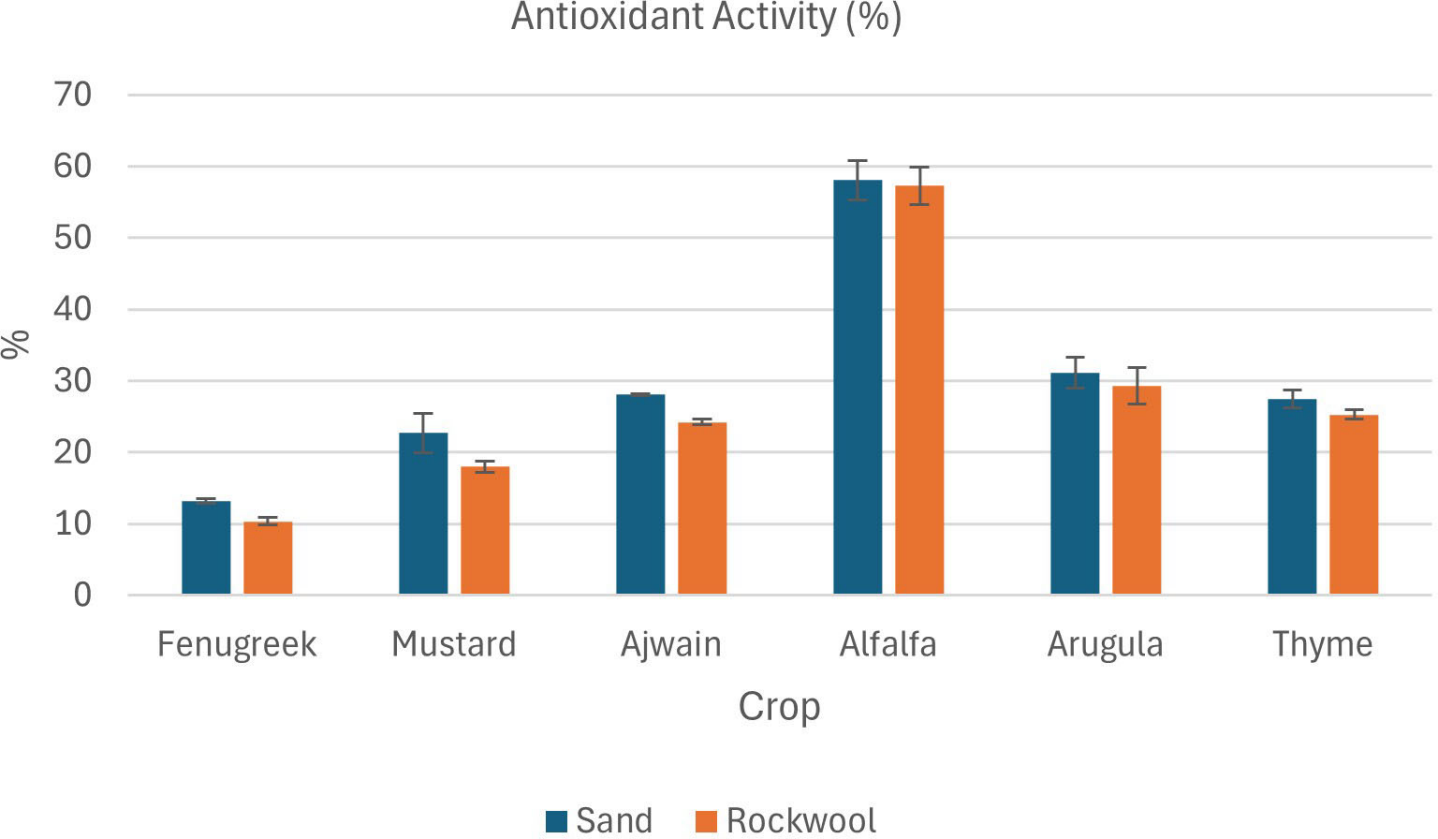
Antioxidant activity (%) in different microgreen species grown under sand and rockwool media. ANOVA with Tukey’s post hoc test was performed (p < 0.05). Different superscripts indicate significant differences; absence of superscripts indicates no significant differences.

Microgreens grown in sand medium displayed higher total antioxidant content compared to those grown in rockwool across all six microgreens (**Figure 8**). There was a significant difference between the total antioxidant content of the microgreens grown in sand and rockwool. Media type significantly influenced DPPH levels with crops grown in sand generally showing higher DPPH content than those grown in rockwool. Crop species also had a highly significant effect on DPPH content, with notable differences observed between various crops. Alfalfa microgreens exhibited the highest total antioxidant content across both growth media. Alfalfa had the highest antioxidant content in sand (58.04%). while the lowest antioxidant content was recorded in fenugreek plants grown in rockwool medium (10.33%). These results clearly demonstrate that both crop species and growth medium strongly affect antioxidant accumulation, and the consistently higher antioxidant content in sand-grown microgreens highlights the potential of sand as a promising substrate for enhancing functional quality.

#### 3.4.4 Total protein content

**Figure 9:**
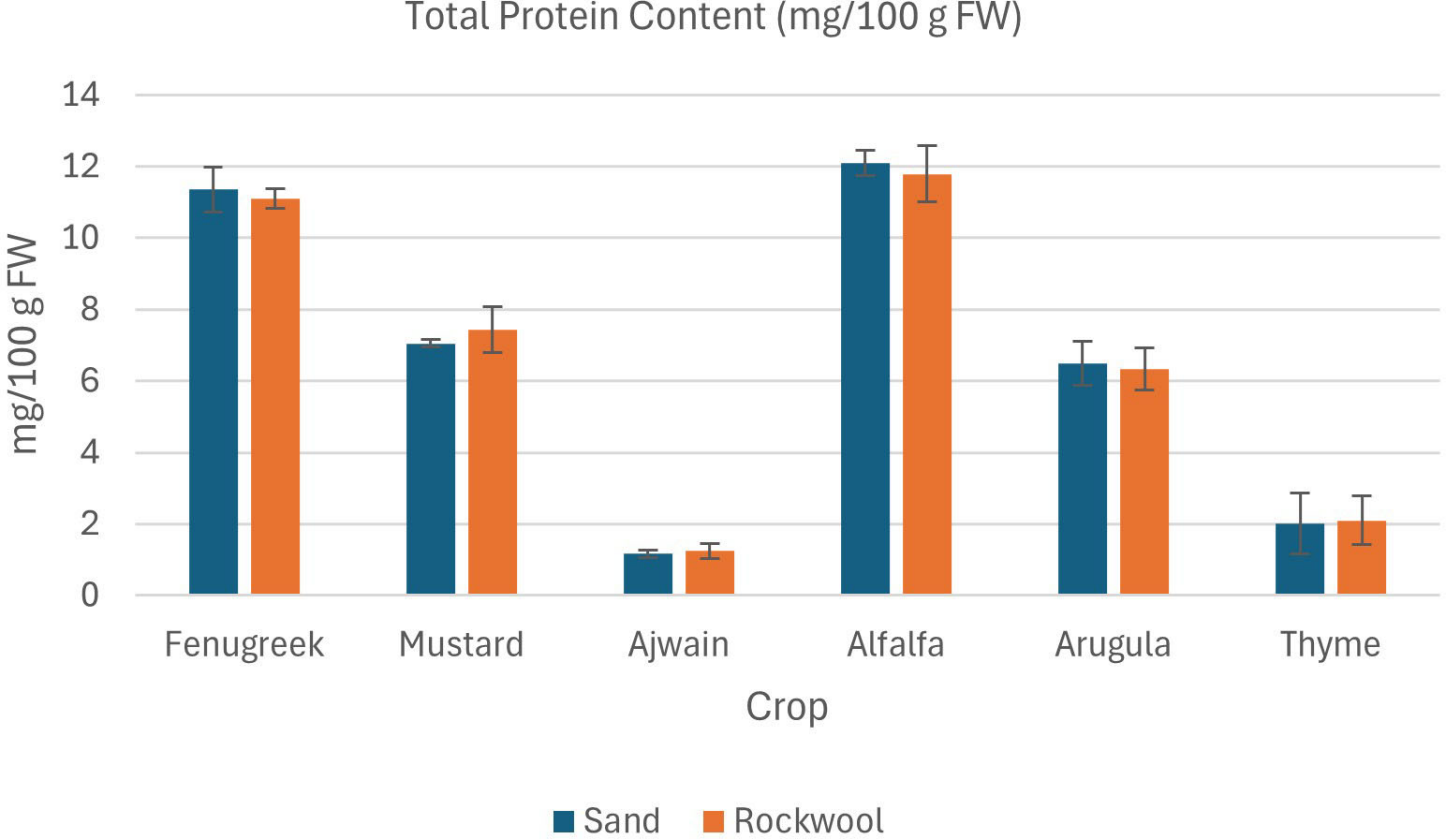
Total protein content (mg/100 g FW) in different microgreen species grown under sand and rockwool media. ANOVA with Tukey’s post hoc test was performed (p < 0.05). Different superscripts indicate significant differences; absence of superscripts indicates no significant differences.

The results of the total protein content in the six microgreen specieswere comparable when grown in both sand and rockwool medium. (**Figure 9**) The results indicate that crop species had a significant influence on protein content, whereas the type of growing medium (sand *vs*. rockwool) had no significant effect. For most crops, the differences between protein levels in sand and rockwool were minimal. Mustard microgreens recorded values of 7.55mg/100g in sand and 7.62mg/100g in rockwool, while fenugreek recordedvalues of 11.5mg/100g in sand and 11.88mg/100g in rockwool. Among the microgreens, alfalfa microgreens grown in sand medium demonstrated the highest total protein content (12.10 mg/100g FW), whereas ajwain microgreens grown in sand exhibited the lowest total protein content (1.16mg/100g FW).

#### 3.4.5 Ascorbic acid content

**Figure 10:**
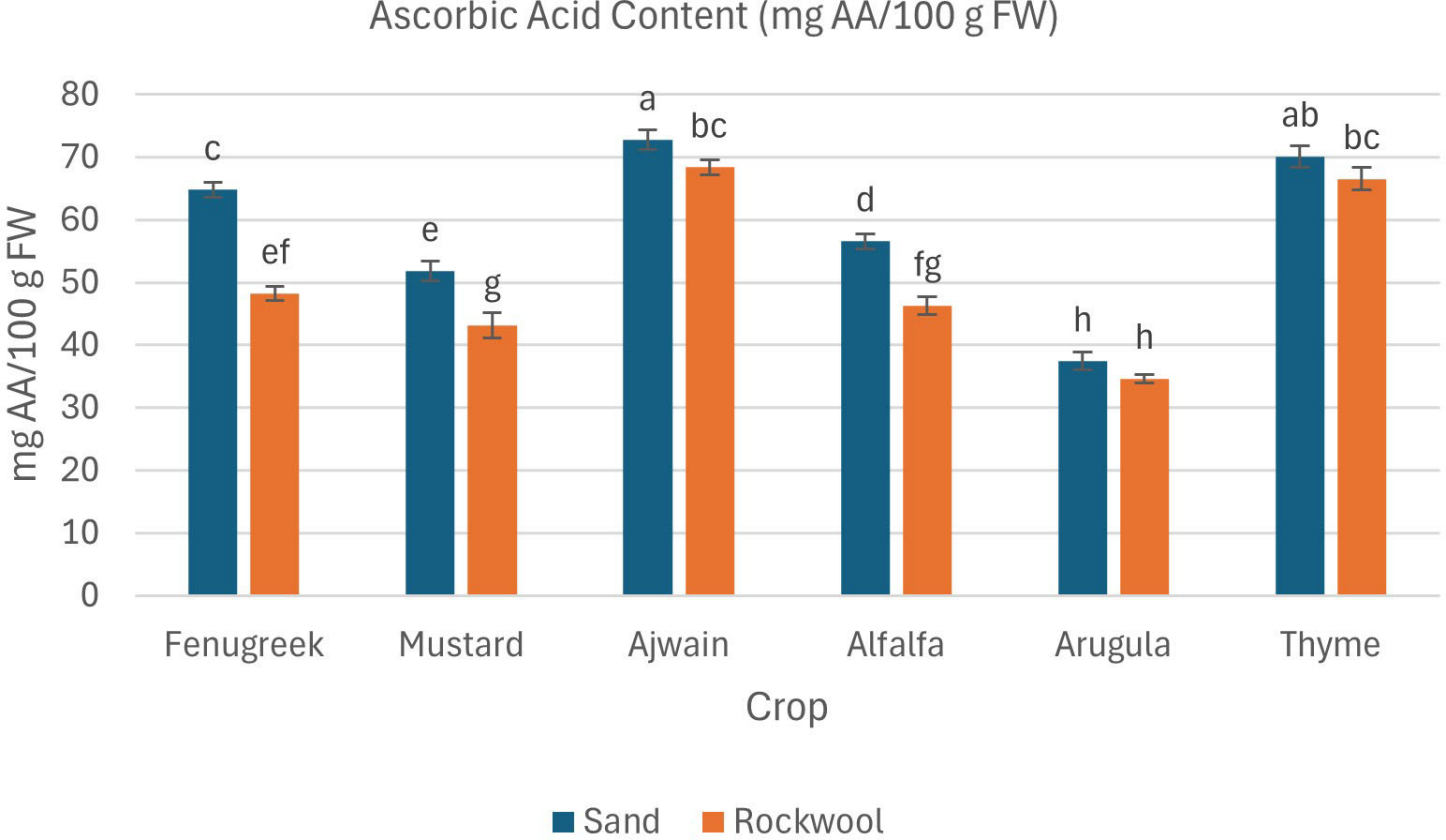
Ascorbic acid content (mg AA/100 g FW) in different microgreen species grown under sand and rockwool media. ANOVA with Tukey’s post hoc test was performed (p < 0.05). Different superscripts indicate significant differences; absence of superscripts indicates no significant differences.

Ascorbic acid accumulation was influenced by both media type and crop species in this study. Microgreens grown in sand generally demonstrated significantly higher levels of ascorbic acid compared to those grown in rockwool (**Figure 10**). Ajwain, fenugreek, mustard, and alfalfa showed significant differences in ascorbic acid content between sand and rockwool, whereas arugula and thyme did not. Among the six species, ajwain consistently exhibited the highest ascorbic acid content, with 72.73 mg AA/100 g FW in sand. The lowest ascorbic acid content was observed in arugula grown in rockwool (34.63 mg AA/100 g FW).

#### 3.4.6 Elemental analysis

The results of elemental analysis is summarized in **Table 5**.

**Table 5:** Elemental analysis of the different microgreen species in sand and rockwool media.

Parameters	Fenugreek		Mustard		Ajwain		Alfalfa		Arugula		Thyme	
	Sand	Rockwool	Sand	Rockwool	Sand	Rockwool	Sand	Rockwool	Sand	Rockwool	Sand	Rockwool
P	26.01 ± 0.72^a^	25.41 ± 0.30^a^	33.43 ± 0.31^a^	30.00 ± 0.66^b^	96.23 ± 1.71^a^	74.87 ± 0.48^b^	108.94 ± 0.84^a^	103.43 ± 2.95^b^	53.90 ± 0.25^a^	50.00 ± 0.76^b^	21.20 ± 0.30^a^	23.43 ± 0.57^a^
K	129.22 ± 0.42^a^	125.59 ± 1.3^a^	308.70 ± 0.55^a^	296.60 ± 0.85^b^	214.07 ± 1.42^a^	193.93 ± 1.42^b^	771.27 ± 0.65^a^	766.10 ± 2.80^b^	320.84 ± 1.38^a^	310.44 ± 0.94^b^	113.68 ± 1.19^a^	110.53 ± 1.69^a^
Ca	51.27 ± 0.35^a^	50.19 ± 0.06^a^	68.20 ± 1.35^a^	68.16 ± 2.70^a^	75.40 ± 1.14^a^	61.27 ± 1.00^b^	133.97 ± 3.27^a^	111.40 ± 1.37^b^	71.47 ± 0.40^a^	69.15 ± 1.58^a^	34.70 ± 1.56^a^	36.61 ± 3.32^a^
Mg	16.33 ± 0.22^a^	14.97 ± 0.21^a^	25.71 ± 0.50^a^	23.27 ± 2.01^a^	18.54 ± 0.89^a^	13.52 ± 1.52^b^	56.04 ± 1.93^a^	54.61 ± 0.88^a^	34.44 ± 1.35^a^	32.60 ± 1.09^a^	9.70 ± 0.55^a^	9.43 ± 0.40^a^
S	17.15 ± 0.60^a^	14.60 ± 0.65^a^	53.05 ± 2.31^a^	59.25 ± 1.73^b^	18.11 ± 1.93^a^	17.72 ± 1.77^a^	21.28 ± 0.54^a^	19.27 ± 0.55^a^	38.28 ± 2.32^a^	37.80 ± 0.36^a^	12.00 ± 0.83^a^	12.59 ± 0.60^a^
Na	7.77 ± 0.15^a^	6.33 ± 0.55^a^	6.60 ± 0.40^a^	7.41 ± 0.35^a^	3.56 ± 0.54^a^	3.06 ± 1.06^a^	7.03 ± 0.47^a^	7.63 ± 0.76^a^	5.59 ± 0.34^a^	7.62 ± 0.28^b^	3.28 ± 0.35^a^	2.40 ± 0.43^a^
Fe	0.92 ± 0.03^a^	0.86 ± 0.06^a^	0.58 ± 0.08^a^	0.46 ± 0.04^a^	0.45 ± 0.02^a^	0.39 ± 0.01^a^	2.60 ± 0.15^a^	2.00 ± 0.20^b^	0.69 ± 0.08^a^	0.60 ± 0.06^a^	0.30 ± 0.02^a^	0.45 ± 0.11^a^
Mn	0.05 ± 0.004^a^	0.05 ± 0.002^a^	0.03 ± 0.005^a^	0.03 ± 0.001^a^	0.03 ± 0.001^a^	0.03 ± 0.005^a^	0.07 ± 0.015^a^	0.06 ± 0.006^a^	0.06 ± 0.007^a^	0.08 ± 0.23^a^	0.019 ± 0.011^a^	0.015 ± 0.001^a^
Zn	0.34 ± 0.01^a^	0.25 ± 0.04^a^	0.39 ± 0.02^a^	0.28 ± 0.02^a^	0.27 ± 0.07^a^	0.22 ± 0.01^a^	0.64 ± 0.09^a^	0.54 ± 0.09^a^	0.31 ± 0.015^a^	0.31 ± 0.05^a^	0.23 ± 0.015^a^	0.22 ± 0.006^a^

Values are given as mean ± SD of 20 samples replicated thrice in each group. Values without superscripts indicate no significant interaction between the factors in the two-way ANOVA analysis. Values that do not share a common superscript (a, b) differ significantly (P ≤ 0.05, Tukey’s HSD test). Differences may be observed between species, substrates, or their interaction as assessed by two-way ANOVA (P ≤ 0.05, Tukey’s HSD test).

Values are given as mean ± SD of 20 samples replicated thrice in each group. Values without superscripts indicate no significant interaction between the factors in the two-way ANOVA analysis. Values that do not share a common superscript (a, b, c, d, e, f) differ significantly (P ≤ 0.05, Tukey’s HSD test). Differences may be observed between species, substrates, or their interaction as assessed by two-way ANOVA (P ≤ 0.05, Tukey’s HSD test)

Microgreens grown in sand medium recorded a higher amount for most of the elements. The amount of phosphorus (P) was highest for alfalfa plants across both substrates and was followed by ajwain plants. Among all macro elements, the amount of potassium (K) exhibited the greatest variation, ranging from a minimum of 110.53 mg per 100 g fresh weight (FW) in thyme grown in rockwool to a maximum of 771.27 mg per 100 g FW in alfalfa grown in sand. The calcium (Ca) content was highest for sand-grown alfalfa plants. A similar trend was also observed in the case of magnesium, wherein alfalfa plants grown in sand recorded the highest concentration (56.04 mg/100g FW). Sodium (Na) amount for sand-grown plants was higher for fenugreek (7.77 mg/100g FW), ajwain (3.56 mg/100g FW) and thyme (3.28 mg/100g FW). The amount of sulphur was highest in mustard and arugula plants belonging to the Brassicaceae family. It was noteworthy to observe that the sand-grown microgreens had a higher concentration of sulphur (S). Also, in both mustard and arugula, the concentration of sodium was higher for the plants grown in rockwool. The micronutrients, Iron (Fe), manganese (Mn) and zinc (Zn) showed only slight variations. The amount of zinc was highest for sand-grown alfalfa plants (0.64 mg/100g FW), while the amount of manganese was highest for rockwool-grown arugula (0.08 mg/100g FW). Iron concentration was highest for alfalfa plants irrespective of the growth medium.

#### 3.4.7 PCA analysis

**Figure 11:**
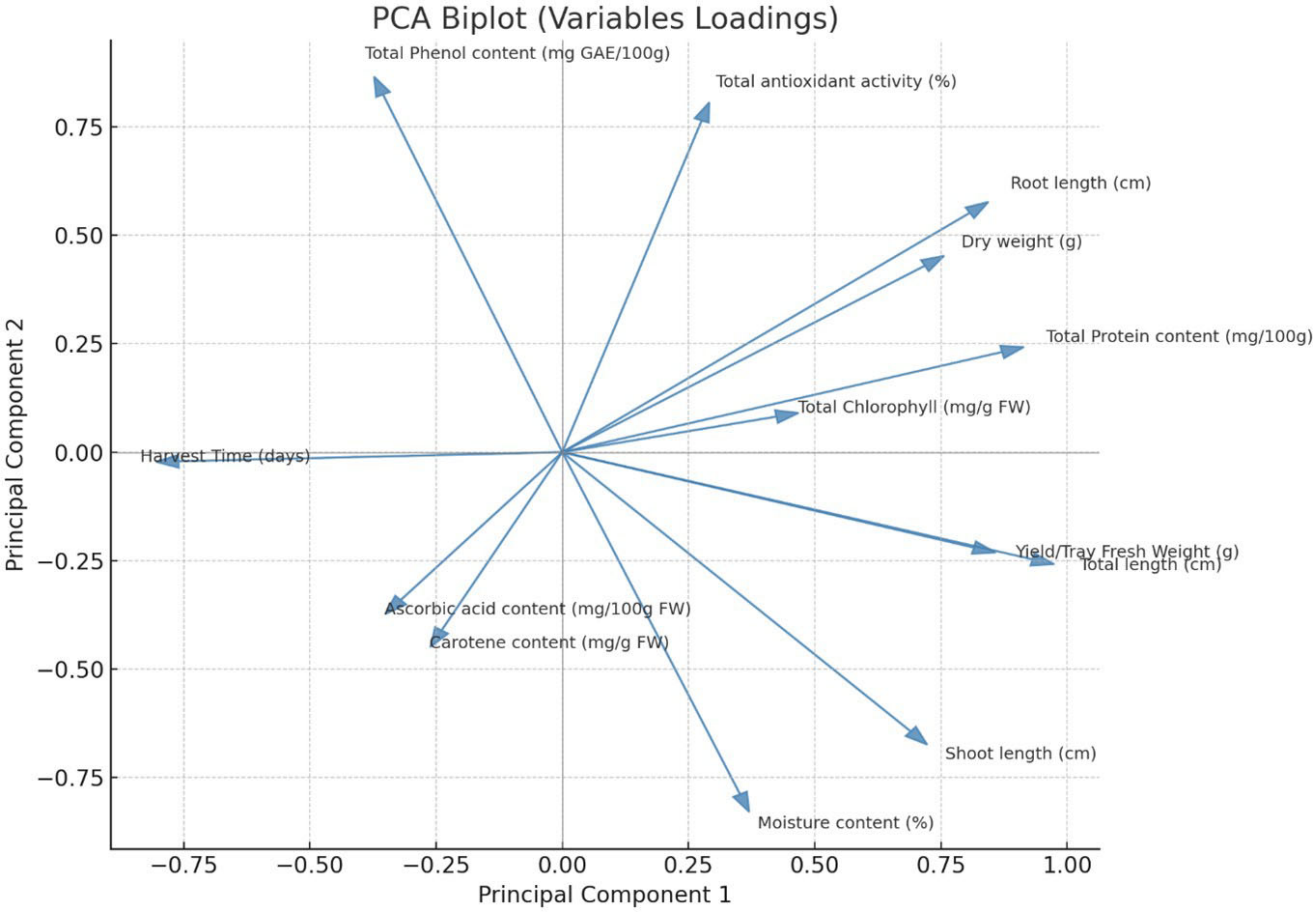
PCA biplot showing variable loadings of growth and biochemical parameters in microgreens.

**Figure 12:**
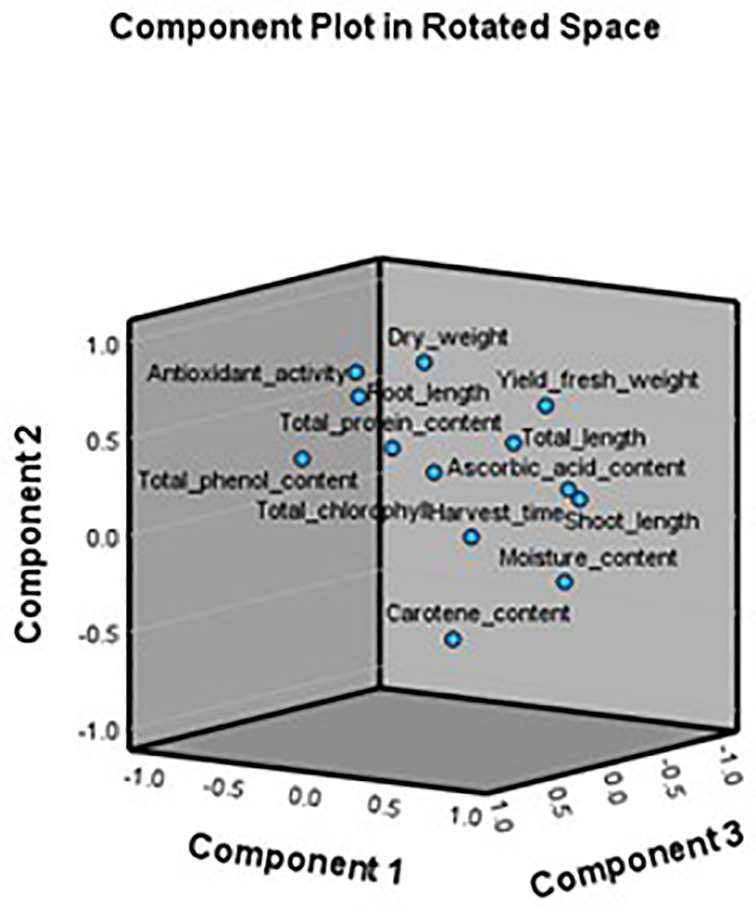
Component plot in rotated space.

PCA Analysis was conducted to understand the relationships among 13 growth and biochemical parameters of 6 microgreens species grown under 2 different substrates (sand, rockwool). This analysis aimed to reduce data dimensionality and identify underlying latent factors. The Kaiser-Meyer-Olkin (KMO) measure of sampling adequacy was 0.749, which indicates that the sample is suitable for PCA. Bartlett’s Test of Sphericity was statistically significant (p<.001), confirming that correlations among variables were adequate to proceed with PCA. The communalities after extraction ranged from 0.750 (Moisture Content) to 0.989 (Root Length), indicating that the retained components explained a substantial portion of variance for most variables. PCA identified four principal components with eigenvalues greater than 1, cumulatively explaining 88.42% of the total variance. Component 1 accounted for 39.82%, Component 2 for 24.98%, Component 3 for 13.65%, and Component 4 for 9.97%. These components were extracted using the Varimax rotation method to enhance interpretability. The rotated component matrix revealed distinct loading patterns. Component 1 showed strong positive loadings for Shoot Length (0.925), Total Length (0.714), Yield/Tray Fresh Weight (0.690), and Moisture Content (0.840). Component 2 was characterized by high loadings for Dry Weight (0.880), Total Antioxidant Activity (0.753), and Root Length (0.750). Component 3 reflected significant contributions from Total Protein Content (0.739) and Root Length (0.603), while Component 4 included high loadings for Total Chlorophyll (0.848) and Carotenoid Content (0.709). The scree plot confirmed the retention of four components, with a clear inflection point after the fourth component. The PCA biplot (**Figure 11**) further illustrates the relationships among variables and components, where related variables are clustered together, while **Figure 12** gives the component plot in rotated space.

## 4 Discussion

### 4.1 Growth parameters

Environmental factors significantly influence plant growth, with key elements such as climate, soil, growing media, watering, light, and seasonal variations playing crucial roles (Kumar and Singh, 2024). Among these, the choice of growing media plays a major role in determining the growth, yield and quality of microgreens and the sustainability of the production process (Benke and Tomkins, 2017; Di Gioia et al., 2017). This experiment evaluates the potential of desert sand as a growth medium for microgreen production by comparing its performance in terms of growth, yield, and quality with that of rockwool. Microgreens generally exhibited better growth parameters when cultivated in sand compared to rockwool, as evidenced by longer shoot and root lengths, and higher yields. In general, all microgreens, irrespective of the type, exhibited enhanced growth parameters in sand, indicating favorable growth conditions in desert sand. The superior performance of desert sand can be attributed to its physicochemical properties and mineral content. In contrast, rockwool possesses high moisture retention capacity which can sometimes lead to anoxic conditions, negatively affecting growth (Blok and Gerard, 2011). Mardiana (2023) also reported better growth in sand compared to high-moisture media like cocopeat.

Variations in growth were observed among different microgreens and substrates(sand, rockwool). These differences are likely due to species-specific physiological characteristics and the composition of the growth media (Saleh et al., 2022). All microgreens adapted well to desert sand, with fenugreek and alfalfa demonstrating superior growth. Similar superior growth of fenugreek compared to other microgreens was also reported by Kalita and Barchung (2022). The effect of medium on root length varied among microgreens, indicating that the interaction between plant species and growth medium affected root development differently. These differences suggest that the choice of growth medium plays a significant role in microgreen development (Di Gioia et al., 2017).

Fresh yield is a crucial factor in microgreen cultivation, particularly since these crops are typically consumed fresh and marketed based on their fresh weight (Jones-Baumgardt et al., 2019). Yield varied between the growth media and microgreen species. Microgreens grown in desert sand generally produced higher yields than those in rockwool, highlighting its potential as a productive growth medium. Despite its relatively low organic matter content, desert sand contains essential minerals and nutrients that may be more readily available to microgreens than in rockwool, supporting robust growth and higher yields. Fenugreek and alfalfa consistently exhibited the highest yields across both substrates, followed by mustard. Variations in yield among microgreens are likely due to differences in growth characteristics, nutrient requirements, and physiological responses to the different growth media. Microgreens grown in desert sand generally exhibited lower moisture content compared to those in rockwool. Desert sand typically has lower water retention capacity compared to rockwool. This is because sand is coarse and porous, allowing water to drain more freely and evaporate quickly In contrast rockwool as a hydroponic substrate has high water retention. It retains moisture for extended periods enabling the microgreens to take up more water. Proper irrigation management can mitigate the limitation of water retention in desert sand. Sinha and Thilakavathy (2021) observed that microgreens grown hydroponically had higher moisture content compared to those grown in other media such as soil. Similar trend was found in dry weights of microgreens grown in sand and rockwool. Higher dry weight in sand grown microgreens might be due to the increased nutrient concentration. Sand, being a natural substrate, contains more minerals and nutrients compared to rockwool. The availability of nutrients in sand can lead to increased nutrient uptake by microgreens, contributing to higher dry weights (Bulgari et al., 2021).

Microgreens cultivated in rockwool reached harvest maturity earlier than those grown in sand. This observation suggested that choice of growth substrate influenced the developmental timeline of these plants. Vakhovska (2021) also concluded from his experiment that the duration of the growth phases of microgreens depended on the substrate. Rockwool provided a controlled environment with consistent moisture and nutrient availability, which might have accelerated the growth rate of microgreens. Due to its variable water retention and nutrient availability, microgreens grown in sand required a longer time to reach harvest maturity compared to those in rockwool, although their overall growth and yield were comparable or higher. In contrast, variable water retention and nutrient availability of sand might have led to slower growth and delayed maturity of microgreens. Each microgreen species has unique physiological characteristics and growth requirements, which can influence the time taken to reach maturity. Factors such as daylight length and temperature also caused variations in harvest time (Vakhovska, 2021). In conclusion, all the results highlighted the positive impact of desert sand as a growth medium for microgreens, particularly in terms of increased growth and yield. The growth and development of microgreens in rockwool depend solely on nutrients available from the water within the rockwool. This can sometimes limit the growth of microgreens after a particular period. Despite the water-retentive properties of rockwool, it did not seem to outperform sand in terms of growth. Overall, the findings suggest that sand is a more suitable growth medium for fenugreek, mustard, ajwain, alfalfa, arugula and thyme microgreens compared to rockwool, as it consistently supported better growth and higher yields.

### 4.2 Phytochemical parameters

Microgreens have been widely studied due to their rich content of vitamins, minerals, and phytochemicals (Lone and Pandey, 2024). In this experiment, all microgreens showed a satisfactory level of phytochemical content with microgreens in sand yielding comparable or slightly higher phytochemical contents compared to rockwool. CChlorophyll content, important for both health benefits and visual appeal, influences consumer acceptance, particularly for color-valued microgreens (Bulgari et al., 2017; Yadav et al., 2019). High chlorophyll levels also highlight their potential as a functional food (Fabek Uher et al., 2023). Among the six microgreen species, mustard consistently exhibited the highest chlorophyll content across both sand and rockwool substrates, indicating its strong photosynthetic capacity and stable chlorophyll synthesis regardless of growth medium. This might be due to the enhanced light capture and energy conversion, highlighting their efficient photosynthetic activity (Ahmed et al., 2025). In contrast, ajwa in microgreens displayed a more pronounced difference in the chlorophyll content between sand and rockwool, indicating a potentially greater sensitivity to substrate variations. This revealed that each microgreen species has unique physiological responses to different growth media. The minor differences in chlorophyll content between sand-grown and rockwool-grown microgreens in fenugreek, alfalfa and arugula suggests that the substrate had minimal influence on chlorophyll synthesis in this species. Chlorophyll synthesis may also be affected by other environmental factors such as temperature, light intensity, water availability (Tripathy and Dalal, 2013). Varying responses of microgreens emphasize the need for further investigation into the specific dynamics between plants and different growth media.

Research has shown that microgreens are a rich source of carotenoids, with varying concentrations depending on the type of microgreen. Xiao et al. (2012) found that different microgreens have varying amounts of carotenoids, with red cabbage, cilantro, garnet amaranth, and green daikon radish having the highest concentration. In this study, while most microgreens cultivated in sand and rockwool displayed similar carotenoid content, ajwain microgreens exhibited a distinct response, with significantly higher carotenoid content observed in sand-grown plants. The results exhibited were in contrast to this with rockwool grown plants recording higher carotenoid content. These findings highlight the importance of considering species-specific responses and substrate influences when assessing carotenoid accumulation in microgreens. Further research is warranted to elucidate the underlying mechanisms driving the observed variations in carotenoid content among different microgreen species and growth media.

Research on microgreens has shown that they are rich in total phenolic content, which contributes to their high antioxidant potential and health benefits (Dhaka et al., 2023). However, the specific phenolic content varies between different species and growth conditions (Kyriacou et al., 2019, 2020). Microgreens cultivated in sand demonstrated a higher total phenol content compared to those grown in rockwool, indicating a potential influence of the growth substrate on phenolic compound accumulation. The observed differences in total phenol content highlights the importance of growth medium selection in modulating the phytochemical profile of microgreens. This observation is particularly significant as phenols are renowned for their role in scavenging free radicals and contributing to the overall antioxidant capacity of microgreens. Dhaka et al. (2023) also reported higher total phenolic content in microgreens. Among the microgreens, sand-grown arugula recorded the highest phenolic content, indicating a potential preference for sand as a growth medium for enhancing phenolic compound production in this species. In contrast, fenugreek had the lowest phenolic content among the six microgreens, especially when grown in rockwool, indicating that the growth substrate may affect the biosynthesis of phenolic compounds differently across species. Studies by Sangwan et al. (2022) also found similar values of total phenol content.

Microgreens are abundant in antioxidants like glucosinolates and phenolic compounds, which are associated with numerous health benefits. Research has shown that these compounds can help boost the immune system, particularly in the prevention of illnesses like COVID-19 (Tallei et al., 2024; Rohmanna et al., 2023). The antioxidant content of microgreens can vary based on factors such as species, growing conditions, and harvesting time (Tallei et al., 2024). Studies by Ghoora et al. (2020) and Dhaka et al. (2023) reported high values of DPPH activity in microgreens. The antioxidant potential was examined in terms of radical scavenging activity (DPPH). In the DPPH assay, free radicals containing DPPH react with sample antioxidants. The antioxidants then reduce the DPPH molecule by donating a hydrogen atom (Senevirathne et al., 2019 **).** Microgreens cultivated in sand exhibited elevated total antioxidant content compared to those in rockwool. This might be due to the effect of salinity from sand on antioxidant properties of microgreens. According to Islam et al. (2019), moderate salinity levels improved antioxidants in microgreens. Among the microgreens, alfalfa had the highest antioxidant content in sand, while fenugreek exhibited the lowest antioxidant content when grown in rockwool. This indicated potential differences in antioxidant metabolism and accumulation among microgreen species in response to different growth substrates. Antioxidant content of microgreens also depends on plant species, light exposure, salinity stress and the harvesting time (Islam et al., 2019; Mlinaric et al., 2020; Tallei et al., 2024).

Alfalfa and fenugreek microgreens demonstrated consistent total protein levels across both sand and rockwool substrates as the microgreens from Fabaceae family are generally rich in protein (Kowitcharoen et al., 2021). Fenugreek possesses inherent physiological mechanisms that enable stable protein biosynthesis regardless of the growth medium resulting in higher protein content. The detected values for fenugreek were higher than the values reported by Ghoora et al. (2020). The variation in the protein content of other microgreen species maybe from species-specific responses to distinct growth media, indicating differential nutrient uptake and utilization pathways among microgreen species. These findings suggest that substrate composition may influence protein accumulation in microgreens, to a lesser extent compared to species-specific factors.

The most valuable antioxidant for living beings, including plants, is ascorbic acid (vitamin C). During various physical or physiological stresses, the free ascorbic acid oxidizes into dehydroascorbic acid and protects from oxidative damage to plants as well as human beings (Gonzalez et al., 2008). Microgreens cultivated in sand generally exhibited higher levels of ascorbic acid compared to those grown in rockwool. According to Saleh et al. (2022) the interaction of the growing media and plant species can be closely associated with microgreens and total ascorbic acid content. Sand, being a natural medium rich in minerals and nutrients, may provide favorable conditions for enhanced biosynthesis and accumulation of ascorbic acid in microgreens. Ajwain microgreens consistently exhibited the highest ascorbic acid content among the six species, irrespective of the growth medium. This observation suggests inherent differences in ascorbic acid metabolism and accumulation pathways among microgreen species. Further investigation is necessary to elucidate the underlying mechanisms governing ascorbic acid metabolism in microgreens. Xiao et al. (2019) and Dhaka et al. (2023) evaluated ascorbic acid in different microgreens and found similar results.

Microgreens, which are the edible young leaves of different plants, are gaining attention as a nutrient-packed crop that could help combat mineral deficiencies. Research shows that microgreens can be a good source of important minerals like potassium (K), calcium (Ca), iron (Fe), zinc (Zn), and copper (Cu) (Di Gioia et al., 2023; Lenzi et al., 2019). Studies by Othman et al. (2020) have shown that microgreens often have significantly higher concentrations of essential nutrients compared to their fully grown counterparts. These nutrients include phosphorus (P), potassium (K), sulfur (S), magnesium (Mg), manganese (Mn), copper (Cu), zinc (Zn), and iron (Fe). This high nutrient density is because microgreens are harvested at a very young stage, just after the first true leaves have developed, when the plant’s nutrient content is typically at its highest. In this study, phosphorus concentration was highest for the sand grown alfalfa microgreens. The stage of development at harvest can greatly affect nutrient composition, with microgreens typically accumulating more phosphorus than baby greens (El-Nakhel et al., 2021). Out of all the macro elements, potassium (K) showed the maximum variation among all the microgreen species. The elemental composition is species specific and is influenced by genetic factors and growth conditions (Mohanty et al., 2021). Genotypic variations in mineral content have been identified among different microgreen species, with potassium (K) and nitrogen (N) being the dominant macronutrients (Di Gioia et al., 2023). The growth media can influence the nutritional content of microgreens (Weber, 2016). Sand being a natural growth medium, can influence the synthesis of elements in microgreens. Thus, the microgreens grown in sand media recorded high elemental concentrations. The microgreens species, mustard and arugula, belonging to the family, Brassicaceae, recorded the highest concentration of sulphur. Brassicaceae microgreens are nutrient-rich and sustainable foods, abundant in bioactive compounds such as glucosinolates, phenolics, and vitamins (Zeng et al., 2023; Alloggia et al., 2023). Sulphur, a vital element for plants, plays a key role in the synthesis of glucosinolates, which are known for their cancer-preventive properties in cruciferous vegetables (Zeng et al., 2023). Similar trends were also observed in the case of micronutrients such as Iron (Fe), Manganese (Mn) and Zinc (Zn) where the sand grown microgreens recorded higher amounts than microgreens grown in rockwool. We can conclude that the influence of growth medium on micronutrient synthesis in microgreens may be more significant than species-specific interactions.

### 4.3 PCA analysis

The PCA results provide insights into the underlying structure of growth and phytochemical traits in microgreens under different substrate conditions. The four extracted components represent distinct latent dimensions.

Component 1, accounting for the highest variance, can be interpreted as a “Growth and Biomass” factor. It encompasses key morphological traits such as Shoot Length, Total Length, and Yield, along with Moisture Content. This component reflects overall vegetative vigor, suggesting that these traits are interrelated and may respond similarly to substrate influences. Component 2 appears to represent a “Physiological and Antioxidant Activity” dimension. High loadings from Dry Weight and Total Antioxidant Activity, along with Root Length, suggest a grouping of traits associated with resource allocation and oxidative stress responses. The co-loading of Dry Weight and Root Length may also indicate efficient water and nutrient uptake mechanisms. Component 3, with strong contributions from Total Protein and Root Length, could be seen as a “Nutritional Quality and Root Function” axis. It implies that protein synthesis may be linked to below-ground development, which could be influenced by substrate type and nutrient availability. Component 4 highlights the “Photosynthetic Pigment and Phytochemical Content” cluster. Traits such as Total Chlorophyll and Carotenoid Content show high loadings, emphasizing species-specific photosynthetic and nutritional traits rather than substrate effects. A previous study by Gunjal et al. (2024) used PCA analysis to show that the growing medium (soil *vs*. cocopeat) significantly affected the morphological, nutritional and bioactive properties of selected microgreens. The presence of high antioxidant and phenolic content in separate components further supports the idea that nutritional enhancement may not necessarily correlate with biomass accumulation. In practical terms, these findings suggest that substrate modifications can selectively enhance specific traits such as yield, pigment accumulation, or antioxidant activity. Understanding these latent components is essential for optimizing cultivation protocols in functional crop production. In summary, PCA effectively reduced dimensionality and revealed coherent trait groupings with agronomic and nutritional relevance. This multivariate approach offers a robust framework for future substrate evaluation and cultivar selection in microgreen systems.

A comparative synthesis of all six microgreen species thus highlights distinct differences in growth, yield, and nutritional quality across the two substrates. Fenugreek and alfalfa consistently demonstrated superior growth and yield, followed by mustard and ajwain, while arugula and thyme generally showed lower performance. In terms of nutritional attributes, ajwain excelled in ascorbic acid content, whereas alfalfa and fenugreek were notable for higher protein, phenolic content, and antioxidant activity. Overall, microgreens grown in desert sand tended to perform better across most parameters compared to those grown in rockwool, suggesting that sand is a more suitable substrate for enhancing both growth and nutritional quality.

## 5 Conclusion

Choosing the right growth medium that meets specific growth requirements and gives desired outcomes is crucial in microgreen production. The findings of this experiment collectively suggest that desert sand can serve as a suitable medium for the growth of microgreens. However, the observed variability among growth and nutritional parameters highlights that the response of each species to the medium should be considered. Desert sand demonstrated potential as a growth medium for microgreens. Further validation will help to confirm its suitability for commercial production. Desert sand showed potential as a microgreen growth medium, promoting longer shoots, better root development, higher yields, and enhanced phytochemical content compared to rockwool. Its greater nutrient availability may also enhance plant metabolism and overall growth. These findings suggest that desert sand is a feasible, low-cost alternative, particularly in arid regions where conventional substrates are limited. However, further research is needed to optimize cultivation practices and evaluate the long-term sustainability and scalability of its use.

## Data Availability

The original contributions presented in the study are included in the article/supplementary material. Further inquiries can be directed to the corresponding author.
